# Development of γ-Oryzanol- and Tocopherol-Enriched Sunflower Oil from Rice Husk via Supercritical CO_2_ Extraction

**DOI:** 10.3390/foods15213850

**Published:** 2026-10-28

**Authors:** Shabnam Haghighat Khajavi, Mutasem Razem, Aparnadevi Premkartikkumar Geethalakshmi, Rajat Suhag, Sara Bolchini, Maria Concetta Tenuta, Edoardo Longo, Emanuele Boselli, Giovanna Ferrentino

**Affiliations:** 1 Faculty of Agricultural, Environmental and Food Sciences, Free University of Bolzano, Piazza Università, 1, 39100 Bolzano, Italy; sh.h.khajavi@gmail.com (S.H.K.); mutasem.razem@unibz.it (M.R.); aparnadevi.premkartikkumargeethalakshmi@student.unibz.it (A.P.G.); rajat.suhag@unibz.it (R.S.); sara.bolchini@unibz.it (S.B.); edoardo.longo@unibz.it (E.L.); emanuele.boselli@unibz.it (E.B.); 2 Department of Food Science and Technology, SR.C., Islamic Azad University, Tehran 1477893855, Iran

**Keywords:** antioxidant recovery, agro-industrial by-products, green extraction, response surface methodology, oxidative stability, thermal processing, circular bioeconomy

## Abstract

Rice husk is an abundant agro-industrial by-product containing γ-oryzanol, tocopherols, and other antioxidant compounds with potential for food applications. This study investigated a supercritical CO_2_-assisted approach for directly transferring rice husk-derived antioxidants into stripped sunflower oil, using the oil as a food-grade receiving phase. A central composite design was used to investigate the effects of pressure and oil loading on γ-oryzanol recovery, tocopherol content, DPPH radical-scavenging activity, oil yield, and the rate of inhibition (*R_inh_*). Multi-response optimization identified 232 bar and 30 g of stripped sunflower oil as a model-derived candidate condition within the investigated range. Under these conditions, the treated oil contained γ-oryzanol and tocopherols and showed higher assay-measured total phenolic and flavonoid contents than untreated stripped sunflower oil. HS-SPME-GC-MS indicated processing-associated changes in the volatile profile, including an increased signal for tentatively identified benzaldehyde and several oxygenated volatile signals. During heating at 180 °C in the absence of food, γ-oryzanol showed greater retention than total tocopherols, with 77.65% and 40.36% retained after 60 min, respectively. These results demonstrate the feasibility of using SC-CO_2_-assisted treatment to transfer rice husk-derived antioxidant compounds into sunflower oil, providing a potential approach for valorizing rice husk in edible-oil enrichment applications.

## 1. Introduction

The increasing demand for sustainable food production has accelerated the efforts to valorize agricultural by-products into functional ingredients, supporting the transition toward a circular bioeconomy [1]. Among cereal crops, rice (*Oryza sativa* L.) is one of the most extensively cultivated worldwide, generating substantial quantities of residues throughout the milling process [2]. Rice husk, the outer protective layer removed during milling, accounts for approximately 20% of the grain weight, resulting in an estimated annual production exceeding 150 million tons worldwide [3]. Despite its abundance, rice husk remains largely underutilized, with most of it being disposed of through open-field burning, landfilling, or low-value energy applications, creating environmental concerns while overlooking its potential as a source of high-value bioactive compounds. Consequently, the valorization of rice husk into functional ingredients represents an attractive strategy for reducing agricultural waste while generating products with added nutritional and economic value.

Although rice husk is primarily composed of cellulose, hemicellulose, lignin and silica, it also contains numerous phytochemicals with recognized biological activities [4]. In addition to its well-documented phenolic acids and flavonoids, rice husk contains valuable lipophilic antioxidants, including tocopherols and γ-oryzanol. However, the occurrence and recovery of these compounds from rice husk have received comparatively limited attention relative to their extensively studied presence in rice bran and rice bran oil [5]. γ-Oryzanol, a mixture of ferulic acid esters of phytosterols and triterpene alcohols, is of particular interest due to its antioxidant, anti-inflammatory, hypocholesterolemic and cardioprotective properties [6]. Likewise, tocopherols constitute the principal forms of vitamin E naturally present in vegetable oils and are among the most effective chain-breaking antioxidants, delaying lipid oxidation by scavenging lipid radicals and interrupting the oxidative chain reaction [7]. Besides their recognized health benefits, both classes of compounds are widely used to improve the oxidative stability, nutritional quality and shelf life of edible oils and lipid-rich foods, making them highly desirable ingredients for clean-label food formulations.

However, the recovery of these lipophilic antioxidants from rice husk is challenging, as they are entrapped within a rigid lignocellulosic matrix that restricts solvent accessibility and mass transfer. Conventional extraction techniques generally employ organic solvents such as hexane, ethanol or acetone to solubilize these compounds. While effective, these methods often require large solvent volumes, prolonged extraction times and energy-intensive solvent recovery steps, in addition to raising concerns regarding solvent residues and environmental sustainability [8]. These limitations have stimulated growing interest in developing greener extraction technologies capable of recovering thermolabile bioactive compounds while minimizing environmental impact.

Among these technologies, supercritical carbon dioxide (SC-CO_2_) extraction has emerged as one of the most promising alternatives for food applications. Operating above its critical temperature and pressure, carbon dioxide exhibits liquid-like solvating power combined with gas-like diffusivity, allowing efficient penetration into plant matrices under relatively mild processing conditions [9]. Moreover, SC-CO_2_ is non-toxic, non-flammable, recyclable and easily removed after depressurization, producing solvent-free extracts suitable for food applications without exposing sensitive compounds to elevated temperatures [10].

In addition, during SC-CO_2_ extraction, edible vegetable oils can be pumped into the extraction vessel as a food-grade receiving and carrier phase. In this configuration, SC-CO_2_ facilitates the recovery of lipophilic compounds from the solid matrix, while the oil phase receives and retains the extracted constituents, enabling their direct incorporation into an edible lipid matrix. In contrast to conventional modifiers, edible oils not only facilitate the extraction of lipophilic compounds but also act as their final carrier, eliminating the need for downstream solvent evaporation and enabling the direct production of functional ingredients [11]. In this context, stripped sunflower oil offers additional advantages since its endogenous antioxidants are removed prior to extraction, allowing any increase in antioxidant content or activity to be directly attributed to compounds extracted from the plant matrix rather than those naturally present in the oil [12]. Although edible oils have occasionally been employed in supercritical extraction processes [13], their use as a receiving phase for direct enrichment of rice husk antioxidants has not been systematically investigated.

Accordingly, this study aimed to develop an oil-assisted supercritical CO_2_ extraction process to produce γ-oryzanol- and tocopherol-enriched sunflower oil from rice husks. Stripped sunflower oil was used in the extraction vessel as a food-grade receiving and carrier phase for rice husk-derived lipophilic antioxidants. A response-surface methodology was applied to examine the effects of extraction pressure and oil loading on γ-oryzanol concentration, total tocopherols content, DPPH radical-scavenging activity, oil recovery yield, and the rate of inhibited oxidation. Numerical multi-response optimization was used to identify a model-derived candidate condition within the investigated design space. The resulting treated oil was subsequently characterized for γ-oryzanol, tocopherols, total phenolic and flavonoid-equivalent contents, initial oxidation indices, and volatile-profile changes. Its thermal behavior was further assessed during heating at 180 °C in the absence of food by monitoring γ-oryzanol and tocopherol retention, peroxide value, conjugated dienes, conjugated trienes, and p-anisidine value. Overall, this integrated strategy combines rice-husk valorization, direct oil enrichment, and frying-relevant quality assessment to develop a solvent-free, naturally antioxidant-enriched edible oil.

## 2. Materials and Methods

### 2.1. Materials

Non-pigmented white rice (*Oryza sativa* L., cv. *Vialone Nano*) husk was kindly supplied by Riseria Melotti S.r.l. (Isola della Scala, Verona, Italy). Chromatographic-grade isopropanol, methanol, *n*-hexane, sodium hydroxide, sodium chloride, anhydrous magnesium sulfate, basic alumina, activated carbon, and 2,2-diphenyl-1-picrylhydrazyl (DPPH) were purchased from Sigma-Aldrich (Milan, Italy). Petroleum ether and diethyl ether were also obtained from Sigma-Aldrich (Milan, Italy). Unless otherwise stated, all chemicals and reagents were of analytical grade and purchased from Sigma-Aldrich (Milan, Italy).

### 2.2. Preparation of Rice Husk

Fresh rice husk supplied by Riseria Melotti S.r.l. (Isola della Scala, Verona, Italy) was first rinsed thoroughly with distilled water to remove adhering dust and other surface contaminants. The cleaned husk was then dried in a forced-air oven at 105 °C for 24 h, reaching a residual moisture content of 5 ± 1% measured using a thermobalance. After drying, the material was milled using a laboratory hammer mill (Mill-LM3100, Perten Instruments, Hägersten, Sweden). The particle size distribution of the ground husk was determined using an orbital sieve shaker (Retsch GmbH, Verder Scientific, Haan, Germany), yielding particle sizes ranging from 50 to 1000 μm. Based on preliminary experiments and to ensure uniform packing during supercritical extraction, only the fraction with particle sizes between 250 and 500 μm was selected for subsequent analyses. The characterization of the dried rice husk reported the following structural constituents: 46.7 g/100 g cellulose, 17.7 g/100 g lignin, 5.1 g/100 g hemicellulose, and 6.9 g/100 g silica. In addition, the fiber fractions, which were not additive to the individual structural constituents, resulted in 70.9 g/100 g neutral detergent fiber and 65.8 g/100 g acid detergent fiber.

### 2.3. Stripped Sunflower Oil Preparation

To eliminate interference from the endogenous antioxidants naturally present in sunflower oil and establish a controlled extraction system, antioxidant-depleted (stripped) sunflower oil was prepared according to the method described by [14], with slight modifications. Briefly, 150 mL of sunflower oil was dissolved in *n*-hexane (1:3, *v*/*v*) and subjected to liquid–liquid extraction in a 1 L separatory funnel using 0.1 M NaOH over three successive washing cycles. The aqueous phase was discarded after each wash, and a few drops of 25% (*w*/*v*) NaCl solution were added whenever emulsion formation occurred. The organic phase was subsequently dried over anhydrous MgSO_4_ and filtered before being purified by gravity column chromatography using basic alumina to remove residual polar compounds and naturally occurring antioxidants. To minimize photooxidation during purification, the column was wrapped in aluminium foil, and the eluent was collected in an ice-cooled flask. The purified oil was then mixed with activated carbon (20 g, approximately 20% *w*/*w* relative to the oil) for 45 min under continuous stirring and repeatedly filtered until a clear solution was obtained. The *n*-hexane was subsequently removed at room temperature (25 °C) using a nitrogen-assisted evaporator (MultiVap 8, LabTech, Sorisole, Italy). Nitrogen pressure was adjusted according to the sample volume and evaporation progress to achieve controlled solvent removal while minimizing oil loss. The resulting stripped sunflower oil (SSO), with a recovery yield of approximately 50–60%, was stored in sealed amber vials at −80 °C until further use. The effectiveness of the stripping procedure was confirmed by supercritical fluid chromatography (SFC), verifying the removal of endogenous tocopherols before the extraction experiments.

### 2.4. Supercritical CO_2_-Assisted Extraction of Rice Husk Bioactives into Stripped Sunflower Oil

#### 2.4.1. Supercritical CO_2_ Extraction Conditions

The extraction experiments were conducted using a laboratory-scale supercritical fluid extraction system (Superfluidi S.r.l., Padova, Italy) equipped with a 1 L extraction vessel and two sequential gravimetric separators. A stainless-steel basket (800 mL capacity) was filled with 340 g of rice husk powder (250–500 μm particle size). The stripped sunflower oil was introduced at the bottom of the packed bed to facilitate its contact with the supercritical CO_2_ stream and the rice husk during extraction. The basket was sealed at both ends using porous stainless-steel mesh filters, allowing homogeneous CO_2_ distribution and continuous flow through the rice husk matrix while retaining the solid material.

The extraction temperature was maintained at 40 °C throughout all experiments to ensure supercritical conditions while minimizing potential thermal degradation of the extracted bioactive compounds. Prior to entering the extraction vessel, carbon dioxide was cooled to 4 °C using a refrigerated chiller and subsequently compressed using a high-pressure diaphragm pump (Lewa LDC–M–9XXV1, Milan, Italy). A central composite experimental design was applied to evaluate the influence of extraction pressure (15–30 MPa) and sunflower oil loading (20–50 g) on the recovery of rice husk bioactive compounds. The CO_2_ flow rate was maintained constant at 1 L h^−1^, and each extraction run was performed for 90 min.

#### 2.4.2. Experimental Design by Response Surface Methodology

The effects of pressure and oil co-solvent on the recovery of rice husk-derived bioactive compounds were investigated using response surface methodology (RSM). Experimental design, model fitting and statistical analysis were performed using Design-Expert 7.0.0 software (Stat-Ease Inc., Minneapolis, MN, USA). Based on preliminary experiments, a central composite design (CCD) was employed to evaluate the influence of two independent variables: extraction pressure (X_1_) and amount of sunflower oil loaded into the extraction vessel (X_2_). The ranges investigated were selected as follows: pressure from 15 to 30 MPa and sunflower oil amount from 20 to 50 g, with the intermediate levels set at 22.5 MPa and 35 g, respectively.

The experimental matrix consisted of 11 randomized extraction runs, including three independent center-point extraction runs at 22.5 MPa and 35 g of oil, allowing estimation of pure error and evaluation of linear, quadratic, and interaction effects of the independent variables. Each non-center design condition was performed as one extraction run. For γ-oryzanol, DPPH activity, *R_inh_*, and total tocopherols concentration, duplicate analytical determinations were performed on samples from each extraction run, and their mean value was used as the response in the RSM analysis. Oil recovery yield was determined once per extraction run. Analytical duplicates were used to assess measurement precision and were not treated as independent extraction replicates in the model fitting or ANOVA.

The oil recovery yield was calculated according to the following Equation (1):

(1) $$Oil\;recovery\left(\%\right)=\frac{Weight\;of\;recovered\;enriched\;oil}{Weight\;of\;initial\;loaded\;oil}\times 100.$$

#### 2.4.3. Regression Modelling

The relationship between the independent variables and the measured responses was described using a second-order polynomial Equation (2):

Y = β_0_ + β_1_A + β_2_B + β_11_A^2^ + β_22_B^2^ + β_12_AB + ϵ, (2)

where Y represents the predicted response variable; β_0_ corresponds to the regression coefficient of the intercept; β_1_ and β_2_ represent the linear coefficients of pressure and oil amount, respectively; β_11_ and β_22_ are the quadratic coefficients; β_12_ represents the interaction coefficient between the two independent variables; and ε is the residual error. Regression coefficients were estimated by fitting the experimental data, and the adequacy of each model was evaluated through ANOVA, coefficient of determination (R^2^), adjusted and predicted R^2^ values, and lack-of-fit testing. The experimental unit for the RSM analysis was one extraction run. Therefore, the regression models and ANOVA were fitted to one response value per extraction run, rather than to the individual analytical duplicate measurements. The pure-error estimate was derived from the three independent center-point extraction runs. Model adequacy was assessed using ANOVA, R^2^, adjusted R^2^, predicted R^2^, coefficient of variation, adequacy precision, and lack-of-fit testing. Numerical multi-response optimization was performed using the desirability function in Design-Expert software. Extraction pressure and stripped sunflower-oil amount were constrained within the investigated ranges of 150–300 bar and 20–50 g, respectively. The objectives were to maximize γ-oryzanol concentration, oil recovery yield, DPPH radical-scavenging activity, *R_inh_*, and total tocopherol concentration. The lower and upper limits for each response corresponded to the minimum and maximum values obtained in the experimental design. γ-Oryzanol and total tocopherols concentration were assigned an importance of 5/5, whereas oil yield, DPPH activity, and *R_inh_* were assigned an importance of 3/5. The solution with the highest overall desirability was selected as a model-derived candidate condition for follow-up characterization.

### 2.5. Characterization of Antioxidant-Enriched Sunflower Oil

#### 2.5.1. Quantification of Gamma-Oryzanol and Tocopherols by Supercritical Fluid Chromatography

The concentrations of γ-oryzanol and tocopherols (α-, β-, γ-, and δ-homologues) in the enriched sunflower oils and heated samples were determined using supercritical fluid chromatography coupled with diode array detection (SFC-DAD). Prior to analysis, oil samples were diluted 1:10 (*w*/*v*) in 2-propanol and filtered through 0.45 μm PTFE syringe filters (LLG GmbH, Meckenheim, Germany). Analyses were performed in duplicate.

Chromatographic separations were carried out using a Shimadzu SFC system (Kyoto, Japan) consisting of a CO_2_ pump (LC-30AD SF), a quaternary liquid pump (LC-40D XR), a degasser (DGU403), an autosampler (SIL-40CXR), and a diode-array detector (SPD-M40). Separation was achieved on a Shim-pack UC-NH_2_ column (4.6 × 250 mm, 5 μm; Shimadzu, Kyoto, Japan) using supercritical CO_2_ as the primary mobile phase and methanol as the organic modifier. The injection volume was 20 μL, and the flow rate was maintained at 4 mL/min. The modifier gradient increased from 5 to 30% over the first 7.5 min, was held at 30% for 0.5 min, decreased to 5% between 8 and 9 min, and remained constant for an additional minute to re-equilibrate the column.

The column oven was maintained at 40 °C, while the back-pressure regulator was set at 15 MPa and 50 °C. The SFC pump head was maintained at 5 °C. Detection wavelengths were set at 330 nm for γ-oryzanol and 290 nm for tocopherols. Quantification was performed using external calibration curves prepared from analytical standards. Instrument control, data acquisition, and chromatographic processing were carried out using LabSolutions software (version 5.127 SP1, Shimadzu, Kyoto, Japan).

#### 2.5.2. Determination of Antioxidant Activity by the DPPH Radical-Scavenging Assay

The antioxidant activity of the enriched sunflower oils was evaluated using the DPPH radical-scavenging assay according to Prevc et al., with minor modifications [15]. A 2.5 mM DPPH stock solution was prepared in isopropanol and diluted to obtain a working solution of 200 μM. Oil samples (50 mg) were dissolved in 450 μL of isopropanol, and 100 μL of the resulting solution was mixed with 1.9 mL of the DPPH working solution. A reagent blank was prepared by replacing the oil sample with isopropanol. The reaction mixtures were incubated in the dark at 25 °C for 30 min before measuring the absorbance at 515 nm using a Cary 60 UV–Vis spectrophotometer (Agilent Technologies, Santa Clara, CA, USA).

Antioxidant activity was expressed as mg Trolox equivalents per 100 g of oil (mg TE/100 g oil), using a Trolox calibration curve prepared under the same assay conditions. Measurements were performed in triplicate.

#### 2.5.3. Oxidative Stability by Isothermal Calorimetry

The oxidative stability of the enriched sunflower oil samples was evaluated by isothermal calorimetry at 40 °C using a Thermal Activity Monitor (TAM III Model 421, TA Instruments, New Castle, DE, USA), following the methodology described by [16]. To ensure a controlled and reproducible oxidation rate, the radical initiator 2,2′-azobis(2-methylpropionitrile) (AIBN) was incorporated into each sample at a final concentration of 25 mM. Briefly, an AIBN stock solution was prepared by dissolving 500 mg of AIBN in ethyl acetate to a final volume of 10 mL. Approximately 82 μL of the stock solution was added to 1 mL of each oil sample, after which the solvent was removed under a gentle nitrogen stream using a Multivap evaporator (Multivap 8, LabTech, Sorisole, Italy). Approximately 100 ± 2 mg of each treated oil sample was transferred into 4 mL glass ampoules, hermetically sealed, and equilibrated inside the calorimeter for 15 min before analysis. Heat flow was continuously recorded at 10 s intervals throughout the oxidation process.

From the calorimetric traces, the conversion fraction (α), a parameter indicating the extent of the oxidation reaction varying from 0 to 1, was calculated based on Equation (3):

(3) α=qtQtot,

where (Q_tot_, Joule) is the overall heat developed during the reaction and q(t) is the cumulative heat flow (expressed in Joules) obtained by integrating the heat flow data. The conversion fraction was used to determine the oxygen consumed during the oil oxidation using Equation (4):

(4) $${\left[O_{2}\right]}_{t}=\left(\frac{V_{H}}{V_{S}}\right)\times \frac{P_{O_{2}}}{R\cdot T}\times \left[1-\alpha (t)\right],$$

where V_H_ (volume of headspace) = 3.7 cm^3^, V_S_ (volume of sample) = 0.3 cm^3^, P_O2_ = 0.21 atm, T = 313 K and R = 0.082 L atm/K mol. From the plot of oxygen consumption vs. time, the rate of the inhibited (R_inh_) period of oil oxidation was determined.

### 2.6. Total Phenolic and Total Flavonoid Contents

Prior to total phenolic content (TPC) and total flavonoid content (TFC) determinations, bioactive compounds were extracted from the enriched oil samples according to Razem et al. [17], with minor modifications. Briefly, 200 ± 1 mg of oil was mixed with 2 mL of *n*-hexane and vortexed for 3 min. Subsequently, 2 mL of 60% methanol was added, and the mixture was vortexed for an additional 3 min to promote partitioning of polar and moderately polar antioxidant compounds into the hydroalcoholic phase. The mixture was centrifuged at 8000× *g* for 5 min at 20 °C to separate the phases. The upper hexane phase, containing the oil fraction, was discarded, while the hydroalcoholic phase was collected and used for subsequent TPC and TFC analyses.

TPC was determined using the Folin–Ciocalteu method described by Margraf et al., with minor modifications [18]. The Folin–Ciocalteu reagent was diluted 1:3 (*v*/*v*) before use. The reaction mixtures were transferred to a 96-well microplate and incubated in the dark for 60 min at 25 °C. Absorbance was measured at 765 nm using an Infinite M Nano+ microplate reader (Tecan, Männedorf, Switzerland). TPC was quantified against a gallic acid calibration curve prepared over the range of 0–400 mg/L and expressed as mg gallic acid equivalents per 100 g of oil (mg GAE/100 g oil). TFC was determined according to Razem et al., with minor modifications [19]. The hydroalcoholic extract was sequentially mixed with sodium nitrite solution (0.5 mol/L), aluminium chloride solution (0.3 mol/L), and sodium hydroxide solution (1 mol/L). The absorbance of the resulting mixture was measured at 415 nm using the same microplate reader at 25 °C. TFC was calculated using a quercetin calibration curve prepared over the range of 0–2000 mg/L and expressed as mg quercetin equivalents per 100 g of oil (mg QE/100 g oil). Both TPC and TFC determinations were performed using four technical replicates, and results were expressed as mean ± standard deviation.

### 2.7. Characterization of Volatile Organic Compounds by Solid-Phase Microextraction Coupled with Gas Chromatography-Time of Flight Mass Spectrometry (HS-SPME-GC-ToF/MS)

Volatile and semi-volatile compounds in stripped sunflower oil and optimized rice husk-enriched sunflower oil were analyzed by headspace solid-phase microextraction coupled with gas chromatography–time-of-flight mass spectrometry (HS-SPME-GC-ToF/MS). For each analysis, 1.0 g of oil was placed in a 10 mL headspace vial, followed by the addition of 1 µL *n*-heptane as the internal standard. The vials were immediately sealed using screw caps fitted with pierceable silicone septa. Sample handling and SPME extraction were performed automatically on a PAL3 autosampler equipped with a cooled sample tray maintained at 10 °C. Before extraction, samples were equilibrated at 40 °C for 15 min under agitation at 250 rpm, applied as alternating cycles of 5 s agitation and 2 s without agitation. Headspace volatiles were extracted at 40 °C for 45 min with a 1 cm DVB/CAR/PDMS SPME fibre (50/30 µm divinylbenzene/carboxen/polydimethylsiloxane; Supelco, Merck Life Science, Milan, Italy). The fibre was then inserted into the GC inlet and thermally desorbed for 6 min at 275 °C.

Chromatographic analysis was performed with a Pegasus BT 4D GC×GC system coupled to a time-of-flight mass spectrometer (LECO, St. Joseph, MI, USA), operated in one-dimensional mode. Separation was carried out on an Rxi-5ms capillary column (30 m × 0.25 mm i.d. × 0.25 µm film thickness; Restek, Bellefonte, PA, USA), using helium as carrier gas at a constant flow rate of 1.0 mL·min^−1^. Samples were analyzed in splitless mode. The septum-purge flow was 6 mL/min, and the inlet purge flow was activated at 6 mL·min^−1^. The GC oven was held at 50 °C for 0.5 min, programmed to 340 °C at 5 °C·min^−1^, and maintained at 340 °C for 1 min. The transfer line and ion source were maintained at 250 °C. Mass spectra were acquired under electron ionization at 70 eV over an *m/z* range of 35–530, at an acquisition rate of 5 spectra·s^−1^ and an extraction frequency of 32 kHz. The ToF instrument was tuned before each analytical sequence. Chromatograms were processed by deconvolution using ChromaTOF^®^ software (version 2021; LECO Corporation, Berlin, Germany). Compounds were tentatively identified by comparison of their experimental EI mass spectra with the NIST 2017 mass-spectral database (version 2.3), supported by calculated linear retention indices (LRIs). LRIs were calculated from first-dimension retention times using a C8-C40 *n*-alkane standard series analyzed under the same chromatographic conditions. Untreated stripped sunflower oil and rice-husk-treated sunflower oil were each subjected to duplicate HS-SPME-GC-MS injections. Peak areas are reported as mean values of duplicate technical analytical injections and were used for semi-quantitative comparison of volatile profiles.

### 2.8. Thermal Treatment of Enriched Oil at Frying Temperature (180 °C) in the Absence of Food

The thermal stability of the rice husk-enriched sunflower oil was evaluated under frying-temperature conditions in the absence of food. Aliquots of 1000 ± 1 mg of enriched oil were transferred into individual amber glass vials, which served as independent heating vessels. An oil bath was prepared in a 1 L IKA^®^ heating pot placed on an IKA^®^ RH digital heating plate equipped with an ETS-D5 electronic thermometer (IKA-Werke GmbH & Co. KG, Staufen, Germany). The bath temperature was maintained at 180 ± 1 °C under continuous magnetic stirring to ensure uniform heat distribution. The sample vials were placed separately in a pre-submerged vial holder within the oil bath. At 0, 15, 30, 45, and 60 min, vials were removed, allowed to cool to room temperature, and stored at −80 °C until further analysis. At each heating time, two independently heated oil samples were prepared. γ-Oryzanol and total tocopherols concentrations were determined by SFC for each independently heated sample. K232, K268, PV, and *p*-AV were measured in duplicate by spectrophotometry for each independently heated sample. Duplicate readings were averaged to yield one value per independently heated sample.

### 2.9. Quantification of Antioxidant Depletion During Thermal Treatment

The concentrations of γ-oryzanol and tocopherols in samples collected at 0, 15, 30, 45, and 60 min of thermal treatment were determined by SFC-DAD as described in Section 2.5.1, using external calibration curves prepared from analytical standards. Total tocopherols were calculated as the sum of α-, β-, γ-, and δ-tocopherol homologues, and antioxidant retention was expressed relative to the concentration measured in the unheated oil at time zero.

### 2.10. Determination of Lipid Oxidation Parameters

#### 2.10.1. Peroxide Value

Peroxide value (PV) was determined according to Shantha & Decker [20], with minor modifications. At each heating time, an oil sample (5.0 ± 0.1 mg) was transferred into a Falcon tube containing 9.9 mL of chloroform–methanol mixture (70:30, *v*/*v*) and vortexed. Ammonium thiocyanate solution (50 µL, 30%, *w*/*v*) was added, followed by vortex mixing. Subsequently, 50 µL of iron(II) solution was added, and the mixture was vortexed again. Samples were kept in the dark for 5 min at room temperature, after which absorbance was measured at 500 nm using a Cary 100 UV–Vis spectrophotometer (Agilent Technologies, Milan, Italy).

A reagent blank containing all reagents except oil was prepared in parallel. PV was calculated from an iron(III) calibration curve prepared over the range 1–100 µg/mL and expressed as milliequivalents of oxygen per kilogram of oil (meq O_2_/kg oil). Analyses were performed in duplicate. This method is based on the oxidation of ferrous ions by lipid hydroperoxides to ferric ions, followed by formation of a coloured ferric thiocyanate complex measured spectrophotometrically at 500 nm.

#### 2.10.2. Conjugated Dienes and Trienes

Conjugated dienes and trienes were determined spectrophotometrically according to Zeb and Haq (2018) [21]. Oil sample (0.005 g) was dissolved in 6 mL of n-hexane, and absorbance was measured at 232 and 268 nm using a Cary 100 UV–Vis spectrophotometer (Agilent Technologies, Milan, Italy). Measurements were performed in duplicate.

The specific extinction coefficients at 232 and 268 nm, expressed as $K_{232}$ and $K_{268}$, respectively, were calculated using Equation (5):

(5) $$E_{1\;cm\lambda }^{1\%}=\frac{A\lambda \times 6\times DF}{m}\times 100,$$

where A(λ) is the absorbance of the sample at 232 or 268 nm, 6 is the hexane volume (mL), DF is the dilution factor, and m is the sample mass in grams.

#### 2.10.3. *p*-Anisidine Value

The *p*-anisidine value (*p*-AV) was determined according to IUPAC Method 2.504 and AOCS Official Method Cd 18–90. The oil sample (0.5–4.0 g) was dissolved in 25 mL of *n*-hexane or isooctane. The initial absorbance of the oil solution A_b_ was measured at 350 nm against the solvent blank. Subsequently, 5 mL of the oil solution was mixed with 1 mL of p-anisidine reagent, prepared by dissolving 0.25 g of p-anisidine hydrochloride in 100 mL of glacial acetic acid. After incubation for exactly 10 min, the absorbance of the reaction mixture A_s_ was measured at 350 nm using a mixture of 5 mL of solvent and 1 mL of *p*-anisidine reagent as the reference blank.

The *p*-anisidine value was calculated according to Equation (6):

(6) $$p-AV=\frac{25(1.2\;A_{s}-A_{b})}{m},$$

where *A_s_* is the absorbance after reaction with p-anisidine, A_b_ is the absorbance of the oil solution before reagent addition, and *m* is the mass of oil in grams. The *p*-AV provides an estimate of secondary aldehydic oxidation products, mainly 2-alkenals and 2,4-dienals, formed during lipid oxidation.

#### 2.10.4. Total Oxidation Value

The total oxidation value (TOTOX) was calculated from the peroxide and *p*-anisidine values to provide an integrated index of primary and secondary lipid oxidation, according to Equation (7) [22]:

(7) TOTOX=2×PV+p−AV,

where PV is the peroxide value and *p*-AV is the *p*-anisidine value.

### 2.11. Statistical Analysis

The number of experimental replicates varied depending on the analytical procedure. Data are expressed as mean ± standard deviation, and statistical significance was considered at *p* < 0.05. The experimental design for process optimization and the statistical analyses of the variables were generated using Design-Expert 7.0 software (Stat-Ease Inc., Minneapolis, MN, USA). For response surface modeling, individual experimental runs are reported as single response values, while assay replicates were used to estimate experimental variability and model robustness through ANOVA and regression analysis. For the heating experiment, two independently heated oil vials were analyzed at each sampling time. Data are presented as mean ± standard deviation (*n* = 2). Differences among heating times were evaluated by one-way ANOVA followed by Tukey’s (HSD) test at *p* < 0.05. Because of the limited number of independent heating replicates, these comparisons are interpreted together with the observed response trends. 3D response plots were generated in Design-Expert 13.0 software trial version (Stat-Ease Inc., Minneapolis, MI, USA), and final figures were produced in OriginPro 2021, version 9.8.0.200 (OriginLab Corporation, Northampton, MA, USA).

## 3. Results and Discussion

### 3.1. Optimization of SC-CO_2_ Assisted Extraction of Rice Husk Antioxidants

The effects of extraction pressure and the amount of stripped sunflower oil loaded into the extraction vessel on γ-oryzanol concentration, oil recovery yield, DPPH radical-scavenging activity, rate of oxidation during the inhibited phase (*R_inh_*), and tocopherols concentration were evaluated using a two-factor central composite response-surface design. Results are summarized in Table 1 and Table 2. Each CCD row represents one independent extraction run. The reported values for γ-oryzanol, DPPH activity, *R_inh_*, and total tocopherols were means of duplicate analytical determinations within that run, whereas yield was determined once per run. Accordingly, the analytical duplicates indicate measurement precision but do not provide independent replication of the extraction process. The models differed in adequacy; γ-oryzanol, DPPH and total tocopherols showed relatively strong fit statistics within the design, whereas yield showed significant lack of fit and *R_inh_* had poor predictive performance. The results were therefore discussed response by response, and the numerical condition returned by the multi-response procedure was treated as a candidate rather than a validated optimum.

The γ-oryzanol response was described by a highly significant quadratic model (*p* < 0.05), with R^2^ = 0.99, adjusted R^2^ = 0.98, and predicted R^2^ = 0.93. Both pressure and oil loading significantly affected γ-oryzanol concentration (*p* < 0.05), whereas their interaction was not significant (*p* = 0.1586). The quadratic terms of pressure and oil loading were also significant (*p* < 0.05), indicating a nonlinear response with an optimum within the investigated range. The lack of fit was not significant (*p* = 0.1058), confirming that the quadratic model adequately described the experimental response. Among the experimental runs, the highest γ-oryzanol concentration was obtained with 225 bar and 35 g of oil (69.80 mg/100 g), closely followed by runs 5 and 10 under the same centre-point conditions (68.63 and 68.13 mg/100 g, respectively), whereas the lowest concentration was recorded when 150 bar and 50 g of oil (27.22 mg/100 g) were applied. At both 150 and 300 bar, increasing the oil loading from 20 to 50 g markedly decreased γ-oryzanol concentration. This behaviour likely reflected the combined effects of pressure-dependent extraction and dilution of the extracted compounds in the receiving oil. Since the amount of rice husk was constant and the response was expressed per 100 g of oil, increasing the oil loading distributed the extracted γ-oryzanol over a larger carrier-oil mass. The significant pressure effect was consistent with the increase in SC-CO_2_ density and solvent strength with increasing pressure, which generally enhances the dissolution and transport of lipophilic compounds [23]. However, the lower concentration observed at 300 bar than at 225 bar indicated that solvent density alone did not determine γ-oryzanol recovery. Its extraction also depends on release from the solid matrix, solubility in SC-CO_2_, and partitioning between the supercritical and oil phases. Wang et al. similarly reported a strong pressure dependence for γ-oryzanol recovery during SC-CO_2_ extraction of rice bran [24], while Bitencourt et al. demonstrated that the presence of oil substantially influences γ-oryzanol solubility and extraction behavior [25].

The oil recovery yield was described by a significant quadratic model (*p* < 0.05), with R^2^ = 0.98, adjusted R^2^ = 0.95, and predicted R^2^ = 0.74. Pressure and oil loading significantly affected yield (*p* < 0.05), and their interaction was also significant (*p* < 0.05), indicating that the effect of oil loading depended on the applied pressure. The quadratic pressure term was significant (*p* < 0.05), whereas the quadratic oil-loading term was not significant (*p* = 0.0967). The highest yield was obtained at 225 bar and 35 g of oil (91.00%), while the lowest recovery occurred at 150 bar and 50 g of oil (29.08%). At 50 g of oil, increasing the pressure from 150 to 300 bar increased the yield from 29.08 to 75.22%, whereas the pressure effect was less pronounced at 20 g of oil, demonstrating the significant pressure and oil interaction. This behavior reflected the role of SC-CO_2_ density in enhancing solvating and mass-transfer capacity, together with the influence of oil loading on the effective CO_2_ exposure per unit mass of receiving oil. Increasing pressure improves the ability of SC-CO_2_ to mobilize lipophilic material, whereas increasing the amount of oil at constant extraction conditions reduces the amount of CO_2_ available per unit oil [26]. However, the yield model showed significant lack of fit (*p* < 0.05), indicating that the quadratic equation did not fully describe the experimental response. Although the relatively high R^2^ and adjusted R^2^ indicated a strong fit to the experimental observations, the lower predicted R^2^ (0.74) suggested reduced predictive reliability. The lack of fit may reflect local curvature or process variability not captured by the second-order model, including the complex mass-transfer and oil-displacement behavior within the extraction system. Therefore, the predicted yield response should be interpreted cautiously and verified experimentally.

The DPPH radical-scavenging activity was described by a significant quadratic model (*p* < 0.05), explaining 97% of the measured variance (R^2^ = 0.97, adjusted R^2^ = 0.95, predicted R^2^ = 0.80). Oil loading had a significant effect (*p* < 0.05), whereas linear pressure and the pressure–oil interaction were not significant (*p* = 0.3882 and 0.1578, respectively). Both quadratic terms were significant (*p* < 0.05), indicating an optimum within the investigated range, while the lack of fit was not significant (*p* = 0.2429), confirming acceptable model adequacy. The highest activity was measured at 225 bar and 35 g of oil (1.70 mg TE/100 g), whereas the lowest occurred at 300 bar and 50 g of oil (0.34 mg TE/100 g). The relatively low activity at the higher oil load may reflect a lower concentration of rice husk-derived antioxidants in the larger receiving-oil mass, because the quantity of antioxidants transferred from the constant mass of rice husk may not have increased proportionally with oil loading. Overall, the absolute DPPH activities were relatively low, possibly because the predominantly non-polar SC-CO_2_ oil system preferentially recovered lipophilic antioxidants while providing limited recovery of polar and matrix-bound phenolics responsible for much of the antioxidant activity of rice husk. Indeed, aqueous rice-husk extracts have shown greater DPPH activity, whereas acetone recovered more γ-oryzanol and tocopherols [27]. Moreover, DPPH responses depend on solvent, reaction kinetics, antioxidant structure, and steric accessibility [15,28]; therefore, the low values represent limited radical-scavenging capacity under the selected assay conditions but do not necessarily indicate poor oxidative stability [29].

The *R_inh_* response showed the weakest predictive performance among the investigated responses. Although the quadratic model was statistically significant (*p* < 0.05), R^2^ = 0.89, adjusted R^2^ = 0.78, and predicted R^2^ = 0.03, indicating a substantial difference between the model fit and its predictive capability. Neither pressure nor oil loading had a significant linear effect on *R_inh_* (*p* = 0.5403 and *p* = 0.1033, respectively). In contrast, their interaction was significant (*p* < 0.05), as was the quadratic pressure term (*p* < 0.05), whereas the quadratic oil-loading term was not significant (*p* = 0.0564). The highest *R_inh_* was obtained at 225 bar and 35 g of oil (7.95 × 10^−8^ M s^−1^), whereas the lowest value occurred at 150 bar and 50 g of oil (3.43 × 10^−8^ M s^−1^). The interaction between pressure and oil loading was evident in the experimental data: increasing pressure from 150 to 300 bar decreased *R_inh_* at 20 g of oil, from 6.90 to 5.18 × 10^−8^ M s^−1^, whereas the same pressure increase raised *R_inh_* at 50 g of oil, from 3.43 to 6.98 × 10^−8^ M s^−1^. The significant lack of fit (*p* < 0.05), together with the predicted R^2^ of 0.03, indicates that the model did not reliably predict *R_inh_* values. *R_inh_* represents the oxidation rate during the inhibited phase and therefore reflects the rate of oxygen consumption while antioxidant protection remains active rather than the duration of antioxidant protection. Consequently, a lower *R_inh_* indicates slower oxidation during the inhibited phase, whereas a higher value reflects faster oxygen consumption. Isothermal calorimetry therefore provides *R_inh_* as a complementary kinetic parameter that should be interpreted together with induction time and the subsequent oxidation rate rather than as an isolated measure of oxidative stability [30,31]. The low predicted R^2^ and significant lack of fit suggest that *R_inh_* was influenced by factors not fully captured by pressure and oil loading. This may be associated with the combined concentration, composition, reactivity, and interactions of the different antioxidant compounds transferred into the oil. Therefore, *R_inh_* should be considered primarily a complementary descriptive response rather than a highly reliable standalone predictive parameter.

**Figure 1:**
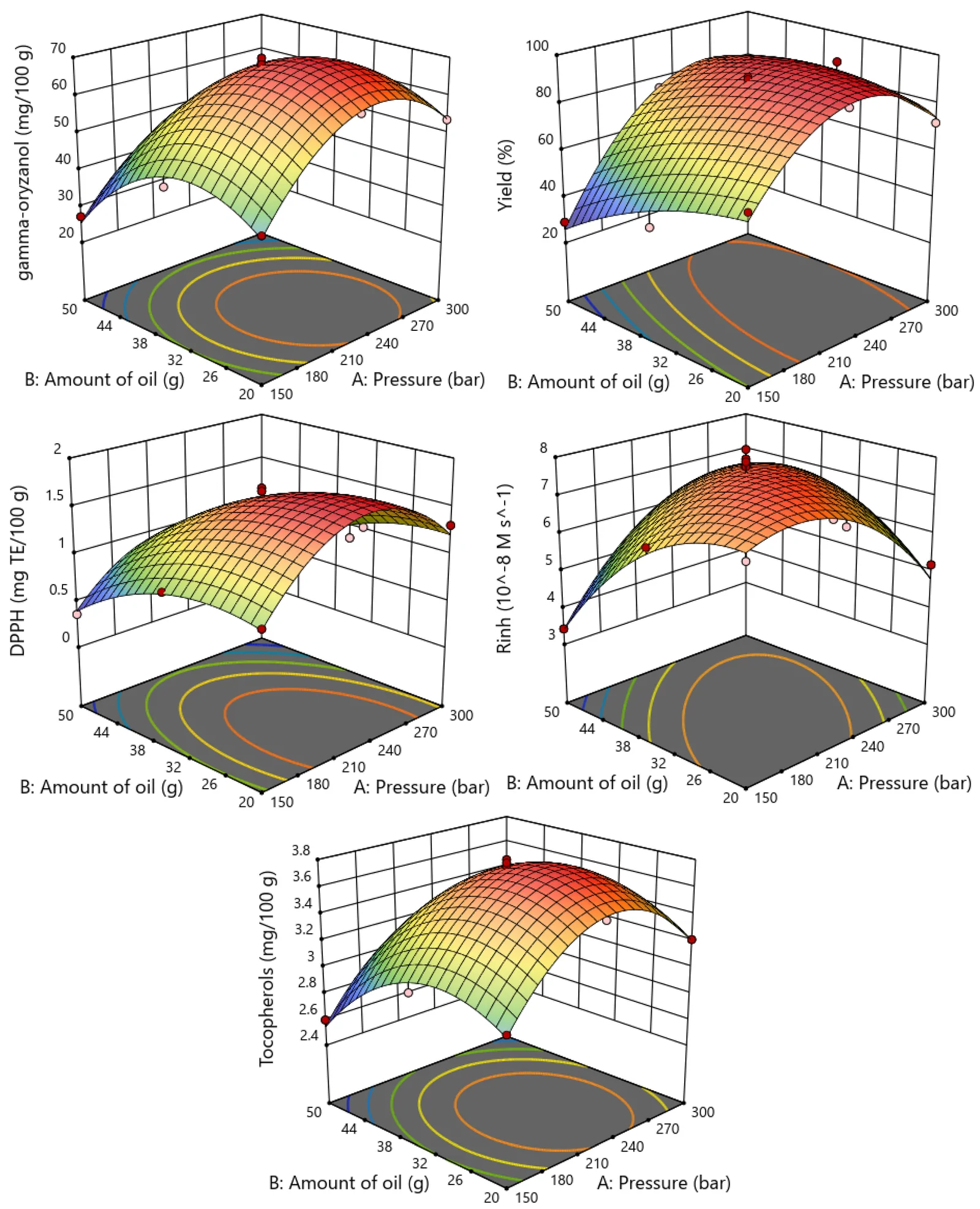
3D response surface plots showing the effects of SC-CO_2_ extraction pressure and stripped sunflower oil loading on gamma-oryzanol content, oil yield, DPPH radical-scavenging activity, rate of inhibition, and tocopherol content.

The tocopherol concentration was described by a highly significant quadratic model (*p* < 0.05), with R^2^ = 0.98, adjusted R^2^ = 0.97, and predicted R^2^ = 0.88. Both pressure and oil loading significantly affected tocopherol concentration (*p* < 0.05), whereas their interaction was not significant (*p* = 0.6571). The quadratic terms of pressure and oil loading were also significant (*p* < 0.05), indicating a nonlinear response with an optimum in the central region of the design space. The lack of fit was not significant (*p* = 0.0600), indicating acceptable model adequacy, although the result was relatively close to the 0.05 significance threshold. The highest tocopherol concentration was measured at 225 bar and 35 g of oil (3.80 mg/100 g), followed by the same center point conditions (3.78 and 3.75 mg/100 g, respectively), whereas the lowest concentration was recorded at 150 bar and 50 g of oil (2.60 mg/100 g). Increasing oil loading generally decreased tocopherol concentration, consistent with dilution of the extracted compounds in a larger receiving-oil phase. The significant pressure effect was consistent with the favorable transfer of lipophilic tocols into SC-CO_2_. Yoon et al. reported that tocols were recovered earlier than γ-oryzanol during fractional SC-CO_2_ extraction of rice-bran oil [32], indicating comparatively favorable solubility and mobility of tocopherols in the supercritical phase. Nevertheless, the lower tocopherol concentration at 300 bar than at 225 bar indicated that increasing solvent density did not continuously improve the concentration of the target compounds. At higher pressure, stronger co-extraction of the bulk oil fraction may reduce the apparent concentration of tocopherols in the recovered phase, contributing to the intermediate optimum observed in the present study. The 3D response surface plots illustrating the combined effects of extraction pressure and stripped sunflower oil loading on the five investigated responses are presented in Figure 1.

The desirability-based multi-response procedure was configured to maximize all measured responses within their observed experimental ranges. γ-Oryzanol and total tocopherols concentration were assigned the highest importance (5/5), whereas yield, DPPH activity, and *R_inh_* were assigned moderate importance (3/5). The numerical solution was 231.93 bar and 29.82 g of oil, rounded to 232 bar and 30 g of oil, with an overall desirability of 0.982. Under these settings, numerical multi-response optimization identified 232 bar and 30 g of stripped sunflower oil as a model-derived candidate condition within the investigated design space. This condition differed slightly from the experimental center point of 225 bar and 35 g of oil, where the highest experimental values were obtained for several individual responses. The difference resulted from the simultaneous optimization of multiple responses rather than maximization of a single response. The selected pressure remained within the region favorable for SC-CO_2_ density and the transfer of lipophilic compounds, while the lower oil loading relative to the center point reduced the extent of dilution of the extracted minor compounds in the receiving oil. This interpretation was consistent with the significant effect of oil loading on γ-oryzanol, DPPH, and tocopherols concentration and with the generally lower values observed at 50 g of oil.

At the candidate condition, the fitted models predicted γ-oryzanol concentration of 69.77 mg/100 g oil, oil yield of 92.54%, DPPH activity of 1.693 mg TE/100 g oil, *R_inh_* of 7.62 × 10^−8^ M s^−1^, and total tocopherols concentration of 3.772 mg/100 g oil. The selected condition should be interpreted cautiously because the yield and *R_inh_* models showed significant lack of fit, and the *R_inh_* model had limited predictive ability. In particular, the yield prediction interval extended above 100%, illustrating a limitation of using an unconstrained polynomial model for this bounded response.

### 3.2. Antioxidant Enrichment Under the Candidate-Derived Condition

#### 3.2.1. γ-Oryzanol and Total Tocopherols

The extraction was performed at 232 bar and 30 g of oil to confirm enrichment with the principal target lipophilic antioxidants and to obtain an oil for subsequent compositional and thermal characterization. The oil obtained under these conditions contained 66.4 ± 1.6 mg γ-oryzanol/100 g oil and 3.4 ± 0.6 mg total tocopherols/100 g. The different behavior of the two compound classes may be related to their distinct extraction and partitioning characteristics. Tocols, particularly α-tocopherol, are more soluble in SC-CO_2_ and are recovered earlier during extraction than γ-oryzanol, which is generally transferred during later extraction stages [32]. In addition, the presence of an oil phase can substantially reduce the apparent solubility of γ-oryzanol in SC-CO_2_, making its recovery more dependent on oil-CO_2_ partitioning and contact conditions [25]. The findings confirmed the enrichment of the stripped oil with the measured lipophilic antioxidants, validating the numerical predictions of the CCD.

#### 3.2.2. Total Phenolic and Flavonoid Contents

In addition to γ-oryzanol and total tocopherols quantification, the enriched oil produced at 232 bar and 30 g of oil showed an increase in total phenolic content (TPC) from 7.4 ± 0.3 mg GAE/100 g oil in the untreated stripped sunflower oil to 15.2 ± 0.2 mg GAE/100 g oil. Total flavonoid content (TFC) also increased from 49.3 ± 0.3 to 61.8 ± 2.5 mg QE/100 g oil. Since the untreated stripped oil was used as the matched control, these increases further indicated that SC-CO_2_ effectively transferred additional antioxidants and flavonoid-equivalent compounds from the rice husk into the oil. These findings show that the optimized process primarily enriched the sunflower oil with lipophilic antioxidants while also increasing its assay-measured phenolic reducing and flavonoid-equivalent contents.

#### 3.2.3. Oxidation Kinetics of the Optimized Oil

The oxidation rate during the inhibited phase of the follow-up extraction of rice husk-enriched sunflower oil was 5.04 × 10^−8^ M s^−1^ ± 5.48 × 10^−9^, which was 33.9% lower than the model prediction of 7.62 × 10^−8^ M s^−1^. This result was also outside the model-reported prediction interval. The discrepancy is consistent with the limited predictive performance of the *R_inh_* model, which showed a significant lack of fit and a low predicted R^2^. As a result, the measured *R_inh_* value was reported as the oxidative stability response of the oil rather than as confirmation of the model-derived candidate condition. This parameter represents the rate of oxygen consumption while the bioactive compounds with antioxidant activity remain active in the AIBN-initiated oxidation system at 40 °C. A lower rate of inhibition indicates a slower oxidation rate during the inhibited period and, therefore, a greater capacity of the bioactive compounds to suppress radical-chain propagation and oil oxidation.

### 3.3. Volatile Profiles of Stripped Sunflower Oil and Rice Husk-Enriched Sunflower Oil

The volatile and semi-volatile profiles of the stripped sunflower oil (SSO) control and the rice husk-enriched sunflower oil (ESSO) were characterized by headspace GC-MS to identify constituents transferred or altered by the supercritical CO_2_ treatment of rice husk. The differences reflect compounds introduced or redistributed by the enrichment process during SC-CO_2_. A total of 26 compounds were tentatively identified based on retention time, linear retention index (LRI), mass-spectral match score, and characteristic quantifier ion (Table 3). Accordingly, the observed differences should be interpreted as changes in the volatile profile that are consistent with, but do not definitely establish, transfer or transformation of rice husk-associated constituents during processing.

The control SSO was dominated by hydrocarbons, alkylated aromatics, and terpenes, consistent with the residual volatile background of refined and stripped vegetable oils [33]. Among the compounds detected in SSO, the largest EIC integrated peak areas were observed for 1-decene (3.6 × 10^8^ count × min), limonene (2.9 × 10^8^ count × min), benzaldehyde (2.6 × 10^8^ count × min), 2-ethyl-1,4-dimethylbenzene (1.7 × 10^8^ count × min), 1-dodecene (1.4 × 10^8^ count × min), 1,2,4,5-tetramethylbenzene (1.4 × 10^8^ count × min), and 1,1′-bicyclohexyl (1.2 × 10^8^ count × min). The tentative detection of limonene agrees with earlier reports identifying terpenes as characteristic minor volatiles of sunflower oil. Bocci & Frega conducted a GC-MS analysis of the volatile fraction of sunflower oil and reported α-pinene as the predominant terpene, followed by limonene, sabinene, β-pinene and camphene [34]. Although the relative abundance observed in the present study differs substantially from that reported by these authors, the presence of limonene supports the occurrence of terpenoid compounds in sunflower oil.

The enrichment process substantially altered the relative volatile profile. The largest change was observed for tentatively identified benzaldehyde, whose EIC integrated peak area increased from 2.6 × 10^8^ count × min in SSO to 9.4 × 10^9^ count × min in ESSO, corresponding to an approximately 36-fold increase, making it the dominant volatile of the enriched oil. Benzaldehyde is a recognized degradation product of phenolic and phenylpropanoid compounds and of phenylalanine, and it is commonly reported in sunflower-based matrices [35]. Its accumulation is therefore consistent with the co-extraction of low-molecular-weight, lignin- and ferulate-derived aromatics from the rice-husk matrix, in line with the total phenolic content and the recovery of γ-oryzanol and tocopherols reported in the preceding sections.

In this context, benzaldehyde may be regarded as an aromatic marker reflecting the transfer of rice husk-derived phenolic constituents into the carrier oil.

In parallel, several tentatively identified oxygenated volatiles absent from the control appeared in ESSO, including hexanoic acid (1.1 × 10^9^ count × min) octanoic acid (1.1 × 10^8^ count × min), nonanal (1.9 × 10^8^ count × min), (E)-2-octenal (1.7 × 10^8^ count × min), 1-octanol (1.1 × 10^8^ count × min), (E)-2-decenal (2.7 × 10^7^ count × min), ethyl decanoate (2.6 × 10^7^ count × min) and 4,6,6-trimethylbicyclo [3.1.1] hept-3-en-2-one (2.2 × 10^6^ count × min). Medium-chain free fatty acids such as hexanoic and octanoic acid are typical products of triacylglycerol hydrolysis and minor lipid breakdown, whereas saturated and unsaturated aldehydes are established secondary volatile markers of lipid oxidation [36]. The appearance of nonanal, (E)-2-octenal, and (E)-2-decenal, derived mainly from the oxidation of oleic and linoleic acid, indicates that the process introduced or concentrated a limited number of oxidation-related volatiles, most plausibly through co-extraction from the plant matrix combined with the oil and CO_2_ contact during extraction [37].

A contrasting trend was observed among several hydrocarbons, alkylbenzenes, and terpenes, which decreased substantially after enrichment. The EIC integrated peak area of 1-decene decreased from 3.6 × 10^8^ to 1.2 × 10^8^ count × min, while limonene decreased from 2.9 × 10^8^ to 2.1 × 10^7^ count × min. Similarly, 1,2,4,5-tetramethylbenzene decreased from 1.4 × 10^8^ to 1.9 × 10^7^ count × min, 2-ethyl-1,4-dimethylbenzene decreased from 1.7 × 10^8^ to 5.8 × 10^7^ count × min, and 1,1′-bicyclohexyl decreased from 1.2 × 10^8^ to 4.2 × 10^7^ count × min. In contrast, 1-dodecene showed an opposite trend, with its EIC integrated peak area increasing from 1.4 × 10^8^ to 3.9 × 10^8^ count × min. These changes indicate that enrichment affected individual volatile constituents differently, rather than producing a uniform decrease in the hydrocarbon and terpene fraction. A few minor constituents shifted only slightly (for example, tridecane from 9.1 × 10^6^ to 8.5 × 10^6^ count × min), while 2,6,10-trimethyldodecane (3.7 × 10^6^ count × min) and 1-tetradecene (4.0 × 10^7^ count × min) were detected only in OSS.

Overall, the HS-SPME-GC-ToF/MS results show that SC-CO_2_ treatment in the presence of rice husk was associated with a substantially changed volatile profile in sunflower oil, characterized by an increased relative response of tentatively identified benzaldehyde and the detection of several oxygenated volatile signals. These results are consistent with transfer, concentration, and/or transformation of volatile constituents during processing, but do not definitively demonstrate the rice-husk origin of individual compounds. The volatile-profile results therefore provide complementary qualitative evidence of compositional change and should be interpreted alongside the measured γ-oryzanol, total tocopherols, and phenolic-equivalent results.

### 3.4. Thermal Stability of Rice Husk-Derived γ-Oryzanol and Tocopherols in Enriched Sunflower Oil Under Frying Temperature Conditions

The thermal stability of rice husk-derived antioxidants in the enriched oil was monitored during heating at 180 ± 1 °C in the absence of food to evaluate their persistence under frying-temperature conditions (Table 4). Because endogenous tocopherols were removed from the sunflower oil before enrichment, as verified by SFC, the tocopherols measured in the enriched oil were attributable to the rice-husk enrichment process. The experiment was not designed as a comparative thermal-stability test because untreated stripped sunflower oil was not heated concurrently. γ-Oryzanol exhibited markedly greater thermal stability than the tocopherols throughout the heating period, retaining 91.3 ± 3.5% of its initial concentration after 15 min and 77.7 ± 3.5% after 60 min. This high retention is consistent with the recognized thermal robustness of γ-oryzanol, whose ferulic acid moiety is esterified to sterols and triterpene alcohols; the conjugated aromatic structure of the ferulate group stabilizes phenoxyl radicals through resonance and confers strong resistance to thermal degradation [38].

In contrast, the total tocopherols degraded more rapidly, decreasing to 71.3 ± 2.2% after 15 min, 49.1 ± 2.8% after 30 min, and stabilizing near 40.4 ± 3.9% after 60 min. This accelerated loss reflects the well-established thermal lability of tocopherols, which act as sacrificial chain-breaking antioxidants that are consumed as they scavenge lipid peroxyl radicals and are additionally susceptible to oxidation and degradation at elevated temperatures [39]. In a recent study of sunflower oil heated under frying conditions, Abrante-Pascual et al. found that tocopherol abundances decreased over time and that the degradation rate of each isoform followed the order of its initial relative proportion, irrespective of reported differences in intrinsic thermal stability [40].

The divergent behavior of tocopherols and γ-oryzanol indicates different contributions to antioxidant protection during heating. Tocopherols act as chain-breaking antioxidants and are progressively depleted through reactions with lipid peroxyl radicals, with substantial losses reported during frying [39]. In contrast, γ-oryzanol can show greater persistence during thermal treatment, although it also undergoes temperature- and time-dependent degradation [38,41]. This persistence may help maintain antioxidant protection during prolonged heating, and γ-oryzanol has previously been shown to improve the thermal and oxidative stability of sunflower oil [42]. In the present study, the greater retention of γ-oryzanol relative to total tocopherols indicates that γ-oryzanol persisted to a greater extent under the applied heating conditions.

### 3.5. Oxidation Progression in Rice Husk-Enriched Stripped Sunflower Oil Under Frying-Temperature Conditions

The rice husk-enriched stripped sunflower oil was evaluated during heating at 180 °C in the absence of food by monitoring conjugated dienes (K232), conjugated trienes (K268), peroxide value (PV), *p*-anisidine value (*p*-AV), and TOTOX value (Table 4). The stripped-oil matrix was selected because endogenous tocopherols were removed before enrichment, allowing the tocopherols and γ-oryzanol detected in the enriched oil to be attributed to rice husk-derived compounds. However, untreated stripped sunflower oil was not heated concurrently. Therefore, the results described the oxidation progression in the enriched oil and did not directly identify an oxidative-stability benefit relative to untreated stripped oil.

K232 increased significantly from 1.97 ± 0.12 at 0 min to 8.21 ± 0.32 after 60 min. Conjugated dienes are formed primarily through the initial oxidation of polyunsaturated fatty acids as double bonds undergo rearrangement during hydroperoxide formation [43]. Their progressive accumulation therefore provides evidence of increasing primary oxidation during the heating treatment. The present pattern is consistent with the expected behavior of sunflower oil, which is particularly susceptible to thermal oxidation because of its high degree of unsaturation [44]. Heating accelerates hydrogen abstraction, oxygen uptake, and formation of lipid hydroperoxides and their associated conjugated structures [45]. The gradual increase in K232 also indicates that the antioxidant compounds introduced during the rice-husk treatment did not completely prevent oxidation under prolonged exposure under the present heating conditions, but rather acted within the oxidative environment generated at 180 °C.

K268 showed a similar evolution, increasing from 1.07 ± 0.07 at the beginning of heating to 3.32 ± 0.11 after 60 min, corresponding to an approximately 210% increase. However, the increase was less clearly resolved statistically than that of K232, with the values measured from 15 to 60 min belonging to the same statistical group. Conjugated trienes are associated with further oxidation and structural rearrangement of polyunsaturated lipid hydroperoxides and therefore provide a complementary indication of thermal oxidation. The simultaneous increase in K232 and K268 demonstrates that oxidation progressed from the formation of primary conjugated structures toward more advanced oxidation products during prolonged heating. Similar increases in conjugated dienes and trienes have been reported for vegetable oils during deep frying, particularly in oils rich in polyunsaturated fatty acids [46].

The PV increased from 0.24 ± 0.06 meq O_2_/kg at 0 min to a maximum of 0.47 ± 0.01 meq O_2_/kg at 30 min, after which it decreased to 0.41 ± 0.01 and 0.31 ± 0.01 meq O_2_/kg at 45 and 60 min, respectively. The initial increase indicates enhanced hydroperoxide formation during the early stages of heating, whereas the subsequent decline is characteristic of thermal oxidation at elevated temperature, where hydroperoxides become unstable and decompose into secondary oxidation products. Thus, the decrease in PV after 30 min should not be interpreted as an improvement in oil quality or reversal of oxidation. Rather, it indicates that hydroperoxide decomposition increasingly exceeded their net accumulation. This decrease coincided with progressive tocopherol depletion, which was consistent with their participation in reactions during thermal oxidation. Alizadeh et al. similarly reported that tocopherol was particularly effective in limiting primary oxidation during deep frying [47]. Thus, tocopherol depletion was consistent with their known susceptibility to degradation and reaction during high-temperature oxidation. As heating continued and the hydroperoxides formed during the earlier stage became unstable, their decomposition led to a decrease in PV, while the increase in *p*-anisidine value indicated the simultaneous formation of secondary oxidation products [48].

The *p*-AV increased continuously and significantly from 7.63 ± 1.59 in the unheated oil to 122.9 ± 0.70 after 60 min, with each heating interval showing a distinct statistical group. This represents the largest relative change among the measured oxidation indices and indicates substantial accumulation of secondary aldehydic oxidation products during prolonged heating. Unlike PV, *p*-AV therefore continued to increase as heating progressed, reflecting the conversion of unstable hydroperoxides into secondary carbonyl compounds. The strong increase in *p*-AV together with the decline in PV after 30 min illustrates the sequential nature of lipid oxidation under the present heating conditions and emphasizes why PV alone is insufficient to characterize the oxidative status of thermally stressed oils.

The TOTOX value also increased during heating, mainly reflecting the pronounced increase in *p*-AV. Because PV remained low and varied only modestly throughout the treatment, the TOTOX trend did not provide substantially different information from *p*-AV under these conditions. Nevertheless, it supported the conclusion that overall oxidative deterioration increased progressively during heating.

Taken together, the measured indices showed progressive oxidation of the rice husk-enriched oil during heating at 180 °C. The data characterized antioxidant retention and the evolution of primary and secondary oxidation products within the enriched formulation. Free fatty acid content or acid value was not determined; therefore, interpretation was limited to antioxidant retention and the selected primary and secondary oxidation indices, while hydrolytic and broader thermal deterioration required further assessment. Plant-derived phenolic compounds can act through hydrogen donation, radical scavenging, metal chelation, and interruption of lipid-radical propagation, although their effectiveness depends strongly on their concentration, chemical structure, polarity, and thermal stability [49,50]. The results are consistent with our previous investigation of Kamut^®^ wheat-bran extract in sunflower oil, in which natural phenolic enrichment reduced conjugated-diene and -triene formation and delayed the development of oxidation products during heating at 180 °C [17]. Moreover, the same sunflower oil and stripping method were used, and peroxide value together with conjugated dienes and trienes were assessed in stripped sunflower oil during heating.

## 4. Conclusions

This study demonstrated the feasibility of valorizing rice husk as a source of naturally occurring antioxidants for direct enrichment of stripped sunflower oil through supercritical CO_2_-assisted extraction, using the oil itself as a food-grade receiving phase. Response surface identified 232 bar and 30 g of oil as a candidate condition for simultaneous optimization within the investigated range. The treated oil contained γ-oryzanol and tocopherols and showed higher total phenolic and flavonoid contents than the untreated stripped-oil control. Since endogenous tocopherols had been removed before treatment, the tocopherols detected in the enriched oil were attributed to the rice-husk treatment. The SC-CO_2_ process also changed the volatile profile, with a marked increase in the signal for tentatively identified benzaldehyde and the appearance of several oxygenated volatiles. These changes support the occurrence of compositional changes during processing. The process enabled enrichment without conventional organic-solvent extraction of the antioxidants or a subsequent solvent-removal step. During heating at 180 °C in the absence of food, γ-oryzanol retained 77.65% of its initial concentration after 60 min, compared with 40.36% for total tocopherols. Meanwhile, the oxidation indices showed progressive oxidation of the treated oil. As untreated stripped sunflower oil was not heated concurrently, further work should compare the oxidative stability against concurrently heated stripped sunflower oil, conventional sunflower oil, and an appropriate antioxidant control. Storage stability, repeated frying with food, sensory properties, free fatty acid content or acid value, and potentially undesirable thermal compounds should also be investigated. Pilot-scale trials, economic and environmental assessments, and further use of the spent rice husk are needed to establish the process’s practical and environmental benefits. Overall, SC-CO_2_-assisted processing offers a potential route for transferring rice husk-derived antioxidants into sunflower oil while valorizing a rice-processing by-product. The results establish the feasibility of antioxidant enrichment and provide a basis for further process development.

## Figures and Tables

**Table 1:** Effects of extraction pressure and oil amount loaded in the vessel on gamma-oryzanol, yield, DPPH radical-scavenging activity, rate of inhibition (*R_inh_*) and tocopherol content of stripped sunflower oil treated with rice husk adsorption combined with supercritical carbon dioxide.

Run	Pressure (Bar)	Amount of Oil (g)	γ-Oryzanol (mg/100 g)	Yield (%, *w*/*w*)	DPPH (mg TE/100 g)	*R_inh_* (10^−8^ M s^−1^)	Total Tocopherols (mg/100 g)
1	300	20	53.64	71.95	1.31	5.18	3.21
2	150	20	41.10	62.79	0.95	6.90	3.01
3	150	50	27.22	29.08	0.36	3.43	2.60
4	225	35	69.80	89.55	1.56	7.95	3.80
5	225	35	68.63	91.00	1.70	7.75	3.75
6	150	35	44.10	41.87	0.95	6.43	3.05
7	300	50	33.03	75.22	0.34	6.98	2.87
8	225	50	42.74	75.76	0.95	5.43	3.08
9	300	35	56.69	87.11	0.87	5.65	3.32
10	225	35	68.13	90.05	1.66	7.88	3.78
11	225	20	62.56	89.86	1.59	6.94	3.56

Note: Each row represents one independent extraction run. Values for γ-oryzanol, DPPH activity, *R_inh_*, and total tocopherols concentration were means of duplicate analytical determinations from the respective extraction run; oil yield was determined once per extraction run. The three center-point rows represented separate extraction runs. TE = Trolox equivalents.

**Table 2:** ANOVA and adequacy statistics for quadratic models describing the effects of SC-CO_2_ pressure and stripped sunflower oil loading on rice husk extraction responses.

Sources of Variation	DF	γ-Oryzanol			Yield			DPPH			Rate of Inhibition			Total Tocopherols		
		Sum of Squares	*f*-Value	*p*-Value	Sum of Squares	*f*-Value	*p*-Value	Sum of Squares	F-Value	*p*-Value	Sum of Squares	*f*-Value	*p*-Value	Sum of Squares	*f*-Value	*p*-Value
Model	5	2208	107	<0.0001	4279	39.22	0.0005	2.33	37.01	0.0006	16.98	7.97	0.0199	1.60	58.25	0.0002
A-Pressure	1	159	38.62	0.0016	1685	77.23	0.0003	0.01	0.89	0.3882	0.18	0.43	0.5403	0.09	16.47	0.0097
B-Amount of oil	1	492	119	0.0001	331	15.15	0.0115	0.80	63.41	0.0005	1.69	3.96	0.1033	0.26	47.07	0.0010
AB	1	11.32	2.74	0.1586	342	15.66	0.0108	0.03	2.76	0.1578	6.94	16.31	0.0099	0.00	0.22	0.6571
A^2^	1	653	158	<0.0001	1497	68.61	0.0004	0.99	78.18	0.0003	3.40	7.98	0.0369	0.60	110	0.0001
B^2^	1	483	117	0.0001	90.94	4.17	0.0967	0.17	13.85	0.0137	2.60	6.11	0.0564	0.32	58.22	0.0006
Residual	5	20.64			109			0.06			2.13			0.03		
Lack of Fit	3	19.16	8.61	0.1058	108	65.93	0.0150	0.05	3.27	0.2429	2.11	68.23	0.0145	0.03	15.82	0.0600
Pure Error	2	1.48			1.09			0.01			0.02			0.00		
Cor Total	10	2229			4388			2.40			19.11			1.62		
Parameters used for adequacy of model																
R^2^		0.99			0.98			0.97			0.89			0.98		
Adj R^2^		0.98			0.95			0.95			0.78			0.97		
Pred R^2^		0.93			0.74			0.80			0.03			0.88		
Adeq Precision		28.25			18.89			16.93			8.70			21.78		
C.V. %		3.94			6.39			10.09			10.18			2.26		

Note: DF: degrees of freedom, C.V.: coefficient of variation.

**Table 3:** Tentatively identified volatile and semi-volatile compounds in stripped sunflower oil (SSO) and rice husk-enriched sunflower oil (ESSO) determined by SPME-GC-MS.

Compounds	Retention Time (min)	Linear Retention Index (LRI)	Quant Mass (*m*/*z*)	Match Score	SSO EIC Integrated Peak Area (Count × min)	ESSO EIC Integrated Peak Area (Count × min)
Benzaldehyde	10.0	926.3	77.1	963	2.6 × 10^8^	9.4 × 10^9^
Hexanoic acid	10.7	942.1	60.0	870	–	1.1 × 10^9^
1-Decene	11.1	953.8	41.1	925	3.6 × 10^8^	1.2 × 10^8^
*p*-Cymene	12.2	992.7	119.1	786	–	1.4 × 10^7^
Limonene	12.3	996.7	68.1	896	2.9 × 10^8^	2.1 × 10^7^
1-Methyl-4-propylbenzene	12.9	1023.4	105.1	770	5.1 × 10^7^	3.6 × 10^7^
(E)-2-Octenal	13.0	1025.6	41.1	878	–	1.7 × 10^8^
1,2,4,5-Tetramethylbenzene	13.1	1029.0	119.1	867	1.4 × 10^8^	1.9 × 10^7^
1-Octanol	13.3	1038.2	41.1	822	–	1.1 × 10^8^
1-Ethyl-2,4-dimethylbenzene	13.7	1053.8	119.1	900	1.0 × 10^8^	3.3 × 10^7^
2-Ethyl-1,4-dimethylbenzene	13.9	1060.7	119.1	913	1.7 × 10^8^	5.8 × 10^7^
Nonanal	14.3	1076.5	57.1	905	–	1.9 × 10^8^
2,5-Dimethyldecane	14.6	1085.9	57.1	782	6.4 × 10^7^	–
Octanoic acid	16.2	1150.4	60.0	853	–	1.1 × 10^8^
1-Dodecene	16.8	1177.0	41.1	934	1.4 × 10^8^	3.9 × 10^8^
Dodecane	17.0	1187.0	43.1	875	7.1 × 10^7^	7.7 × 10^7^
2,2-Dimethyl-1-octanol	17.2	1193.8	70.1	509	–	3.0 × 10^7^
4,6,6-Trimethylbicyclo [3.1.1]hept-3-en-2-one	17.6	1207.1	107.1	853	–	2.2 × 10^6^
(E)-2-Decenal	18.8	1252.7	43.1	763	–	2.7 × 10^7^
2,6,11-Trimethyldodecane	19.3	1269.7	43.1	868	4.7 × 10^6^	4.3 × 10^7^
2,6,10-Trimethyldodecane	19.7	1284.2	43.1	779	–	3.7 × 10^6^
Tridecane	19.8	1289.2	57.1	758	9.1 × 10^6^	8.5 × 10^6^
1,1′-Bicyclohexyl	20.2	1303.0	82.1	891	1.2 × 10^8^	4.2 × 10^7^
1-Tetradecene	22.2	1380.2	41.1	931	–	4.0 × 10^7^
Ethyl decanoate	22.3	1384.4	88.1	719	–	2.6 × 10^7^
Ethyl tetradecanoate	31.5	1780.6	88.1	863	1.5 × 10^7^	1.3 × 10^7^

Note: Compounds were tentatively identified by comparing experimental electron-ionization mass spectra with the NIST 2017 mass-spectral database and by comparing the experimental LRIs with the reference retention-index data. LRIs were calculated against the first-dimension retention times of a C8-C40 *n*-alkane series analyzed under the same chromatographic conditions. EIC integrated peak areas are expressed as count × min; results represent the mean of duplicate analyses.

**Table 4:** Effect of heating time (0–60 min) on retention of γ-oryzanol and total tocopherols, and the primary and secondary oxidation indices of rice husk-enriched sunflower oil produced by supercritical CO_2_ treatment at 180 °C.

Time (min)	γ-Oryzanol Retention (%)	Total Tocopherols Retention (%)	K232	K268	PV (meqO_2_/kg)	*p*-AV	TOTOX Value
0	100 ± 6.03 ^a^	100 ± 15.29 ^a^	1.97 ± 0.12 ^e^	1.07 ± 0.07 ^b^	0.24 ± 0.06 ^c^	7.63 ± 1.59 ^e^	8.12 ± 1.48 ^e^
15	91.34 ± 3.48 ^ab^	71.31 ± 2.21 ^ab^	3.35 ± 0.25 ^d^	2.40 ± 0.23 ^a^	0.30 ± 0.02 ^c^	43.7 ± 0.45 ^d^	44.3 ± 0.49 ^d^
30	88.66 ± 1.46 ^ab^	49.14 ± 2.79 ^b c^	5.05 ± 0.02 ^c^	2.66 ± 0.56 ^a^	0.47 ± 0.01 ^a^	72.0 ± 2.04 ^c^	72.98 ± 2.00 ^c^
45	85.22 ± 0.44 ^b^	40.92 ± 1.80 ^c^	6.75 ± 0.14 ^b^	2.86 ± 0.13 ^a^	0.41 ± 0.01 ^ab^	100.9 ± 1.16 ^b^	101.75 ± 1.19 ^b^
60	77.65 ± 3.52 ^b^	40.36 ± 3.87 ^c^	8.21 ± 0.32 ^a^	3.32 ± 0.11 ^a^	0.31 ± 0.01 ^bc^	122.9 ± 0.70 ^a^	123.52 ± 0.66 ^a^

Note: K232 and K268 represent the specific extinction coefficients associated with conjugated dienes and conjugated trienes, respectively; PV, peroxide value; *p*-AV, *p*-anisidine value; TOTOX value, total oxidation value. γ-Oryzanol and total tocopherol contents are expressed as percentage retention relative to their respective initial concentrations at 0 min, defined as 100%. Results are expressed as mean ± standard deviation (*n* = 2). Within each column, different lowercase superscript letters indicate significant differences among heating times (*p* < 0.05) according to one-way ANOVA followed by Tukey’s HSD test.

## Data Availability

The data supporting the findings of this study are available within the article. Additional information may be obtained from the corresponding authors upon request.
